# Transition of Serum Cytokine Concentration in *Rickettsia japonica* Infection

**DOI:** 10.3390/idr12030023

**Published:** 2020-12-11

**Authors:** Makoto Kondo, Yoshiaki Matsushima, Kento Mizutani, Shohei Iida, Koji Habe, Keiichi Yamanaka

**Affiliations:** Department of Dermatology, Mie University Graduate School of medicine, 2-174 Edobashi, Tsu, Mie 514-8507, Japan; pjskt886@yahoo.co.jp (M.K.); matsushima-y@clin.medi.mie-u.ac.jp (Y.M.); k-mizutani@clin.medic.mie-u.ac.jp (K.M.); kmcasters@yahoo.co.jp (S.I.); habe-k@clin.medic.mie-u.ac.jp (K.H.)

**Keywords:** Japanese spotted fever, *Rickettsia japonica*, IL-6, IFN-γ, eosinophil

## Abstract

(1) Background. *Rickettsia japonica* (*R. japonica*) infection induces severe inflammation, and the disappearance of eosinophil in the acute stage is one of the phenomena. (2) Materials and Methods. In the current study, we measured the serum concentrations of Th1, Th2, and Th17 cytokines in the acute and recovery stages. (3) Results. In the acute phase, IL-6 and IFN-γ levels were elevated and we speculated that they played a role as a defense mechanism against *R. japonica*. The high concentration of IFN-γ suppressed the differentiation of eosinophil and induced apoptosis of eosinophil, leading to the disappearance of eosinophil. On day 7, IL-6 and IFN-γ concentrations were decreased, and Th2 cytokines such as IL-5 and IL-9 were slightly increased. On day 14, eosinophil count recovered to the normal level. The transition of serum cytokine concentration in *R. japonica* infection was presented. (4) Conclusions. IL-6 and IFN-γ seem to be critical cytokines as defense mechanism against *R. japonica* in the acute phase, and this may deeply connect to the decrease of eosinophil.

## 1. Introduction

Japanese spotted fever (JSF) caused by *Rickettsia japonica* (*R. japonica*) infection was first reported from Tokushima Prefecture in Japan in 1984 [1]. The typical symptom of JSF is characterized by diffuse erythema on the whole body, palmar erythema, headache, high fever, and chills after two to eight days following a tick bite [2,3]. Tetracycline is recommended to treat JSF. One of the severest complications is disseminated intravascular coagulation (DIC), low consciousness level, low blood pressure, multiple organ failure, and edema, which occurs if the disease progress is sudden or the diagnosis of JSF is delayed, and some fatal cases have been reported [2,3,4]. Therefore, the deterioration of symptoms following a JSF infection needs to be understood. *R. japonica* infection induces severe inflammation [2,5], and moreover the disappearance of eosinophil in the acute stage of JSF is one of the phenomena. In the current study, we measured the serum concentrations of Th1, Th2, and Th17 cytokines in the acute and recovery stages and discussed the possible immune mechanism in *R. japonica* infection.

## 2. Materials and Methods

This study enrolled 7 patients (3 males and 4 females from 70 to 87 years old, living in the endemic area in Mie Prefecture, Japan) after obtaining written informed consent. The protocol was approved by the Institutional Review Board at Mie University (Tsu, Mie, Japan, permit number 3064). All patients were diagnosed as having JSF by performing PCR for *R. japonica*-specific DNA from blood and/or skin samples [6], or anti-*R. japonica* antibody titer of IgM > 80 in the acute stage, or elevation of IgM and/or IgG more than 4 times in the recovery stage compared to acute stage by indirect immunofluorescence assay (IFA). For this assay slides are coated by Vero cells cultured with *R. japonica* and incubated with the patient’s serum. If antibodies against *R. japonica* are present, the cells fluoresce. 

The serum samples were collected at the first visit (day 1, acute stage) and 14 days after the first visit (recovery stage) and then stored at −80 °C before use. Serum samples were also collected on days 3 and 7 for cases 1–3. We measured cytokine concentrations including Th1 (IFN-γ, IL-12p70, TNF-α, IL-2, IL-8), Th2 (IL-4, IL-5, IL-9, IL-33), and Th17 (IL-6, IL-17A, IL-17F, IL-22). All patients received minocycline on day 1 and recovered on day 14 with no symptoms of JSF and no abnormal data. For cases 1, 2, and 3, the above-mentioned cytokines were measured at 4 points: days 1, 3, 7, and 14. The cytokine measurements were performed with 15 µL of serum for each sample using a panel kit (AimPlex Biosciences, Pomona, CA, USA) and flow cytometer analysis with a BD Accuri C6 device (Becton, Dickinson and Company, Franklin Lakes, NJ, USA) according to the manufacturer’s instructions. We also presented data of white blood cell (WBC), neutrophil, and eosinophil count, as well as C-reactive protein (CRP). The data were measured at the laboratory in our hospital by using an automated indicator. 

Statistical analysis was performed using PRISM software version 6 (GraphPad, San Diego, CA, USA). The Mann–Whitney U test was used to compare cytokine concentrations between days 1 and 14. Differences were considered significant at *p* < 0.05.

## 3. Results

### 3.1. WBC and CRP

**Figure 1:**
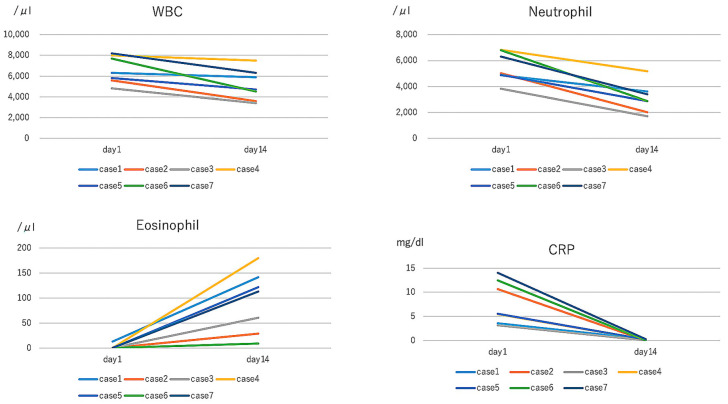
In the acute phase, white blood cell (WBC) and neutrophil were within the normal range, and C-reactive protein (CRP) were increased, but decreased at day 14 by the treatment of minocycline. The loss of eosinophil was detected in the acute stage but recovered to the normal range in all cases.

In all cases, WBC count was elevated in the acute stage and decreased in the recovery phase. The neutrophil count showed similar tendency. Six of the seven patients showed no eosinophil count initially, although one case showed 0.2% in the acute stage, which increased in the recovery stage. CRP was elevated in all cases in the acute stage but decreased to the negative range. All patients responded well without erythema, fever, and fatigue by the treatment of minocycline (Figure 1). 

### 3.2. Inflammatory Cytokine

**Figure 2:**
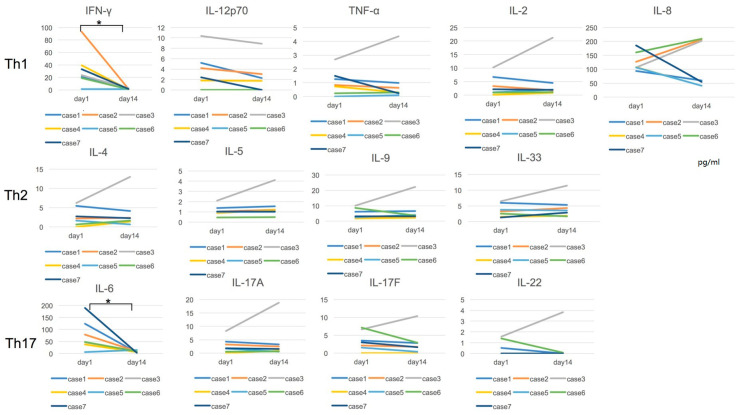
Cytokine concentration in the acute and recovery stages is shown. The increased IFN-γ and IL-6 levels were clearly decreased in the recovery stage in most of the cases. For Th1, Th2, and Th17 cytokines, except for IFN-γ and IL-6, cytokine levels were unchanged for 2 data points (days 1 and 14). Case 3 was exceptional and the cytokines, except for IFN-γ, IL-6, and IL-12p70, were elevated in the recovery stage. Statistical analysis was performed for the cytokine concentration between days 1 and 14, and significance was detected in IFN-γ and IL-6 levels (* *p* = 0.0111).

Among the 13 cytokines that we measured, IFN-γ and IL-6 levels were increased in the acute stage and decreased clearly after the treatment except for cases 3 and 5. Regarding the other measured Th1, Th2, and Th17 cytokines, Th1 cytokines showed a slightly decreased tendency in most of the cases. In contrast, Th2 and Th17 cytokines were unchanged for two data points. In case 3, the counter cytokines against intracellular parasite, IL-6 was undetected at days 1 and 14, but other cytokines were detected (Figure 2). Statistical analysis was performed for the cytokine concentration between days 1 and 14, and significance was detected in IFN-γ and IL-6 levels (*p* = 0.0111).

**Figure 3:**
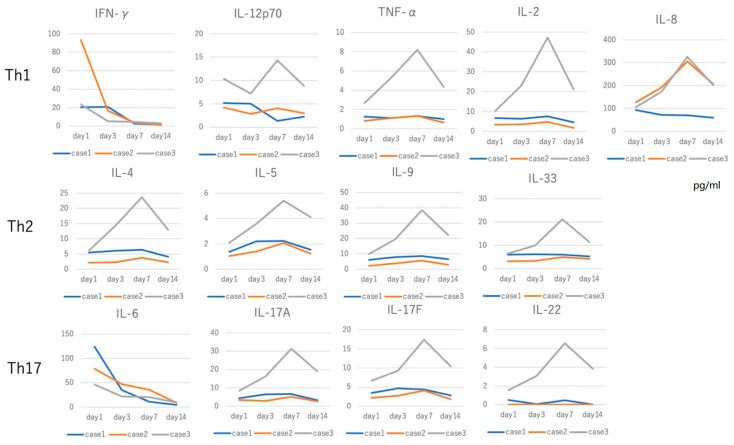
Cytokine concentrations were measured at 4 points in cases 1, 2, and 3. IFN-γ and IL-6 levels showed a peak on day 1, and reduced in a time-dependent manner. IL-5 and IL-9 are involved in the Th2 cytokine families, and the concentrations of those cytokines increased on day 7 and then decreased in the recovery stage. Th17 families except for IL-6 showed a slight peak on day 7. This tendency was not noticed for 2 data points (days 1 and 14).

Cytokine concentration was measured at four points in cases 1, 2, and 3. Among 13 cytokines, elevated IFN-γ and IL-6 levels were clearly decreased over time (Figure 3). Th1 cytokines including IL-12p70, TNF-α, IL-2, and IL-8 did not show this decrease. IL-5 and IL-9 are involved in the Th2 cytokine families, and there seems to be no significance in two points of the acute and recovery stages (Figure 2). However, the concentrations of those cytokines increased on day 7 and then decreased in the recovery stage close to the day 1 level. IL-17A/F also showed a slight elevation on days 3 and 7 (Figure 3).

## 4. Discussion

There has been a report of *R. japonica* infection focused on inflammatory cytokines. In *R. japonica* infection, IL-6, IFN-γ, TNF-α, IL-8, MCP-1a, and MIP-1β levels were elevated according to the previous report [7].

The first line of defense against *Rickettsia* infection is phagocytosis by macrophage, but *Rickettsia* is so-called macrophage-tropic bacteria, and interaction occurs during the phagocytosis, releasing IL-6 and IFN-γ from macrophage as an immune protection. *Rickettsia* existing as an intracellular parasite has the ability to infect the endocapillary cells. Although IL-6 is the key cytokine for the differentiation of Th17 cells, IL-6 was released from the stimulated macrophage-activated Th1 cells during the acute stage of *R. japonica* infection, and Th1 cells produced IFN-γ. IL-6 together with IFN-γ strongly activate macrophage and evoke differentiation of naïve T cells into mature Th1 cells in order to attack pathogens such as *R. japonica* from the outside [8,9].

In *Rickettsia* infection, a high level of CRP was detected, but WBC count showed a slight elevation. A previous report showed that eosinophil counts were diminished similarly to the current cases [10]. Because of the low concentration on day 1 and the increase on day 7 of Th2 cytokine including IL-4, IL-5, and IL-33, we speculated that there may be suppressing factors on eosinophil in *R. japonica* infection, which is purposeful in the acute stage. Th2 cytokines have lower priority because of no necessity of differentiation to eosinophil in the acute phase. The high concentration of IFN-γ suppresses the differentiation of eosinophil and induces apoptosis of eosinophil [11], leading to the disappearance of eosinophil. Around day 7, the number of *R. japonica* is decreased, together with the decrease in IL-6 and IFN-γ concentrations, and the number of eosinophil recovers and Th2 and Th17 cytokines are relatively increased. In the current study, case 3 showed a high concentration in most cytokines measured, although the patient did not have a specific past history such as immunodeficiency, severe allergy, or clinical course associated with multiple organ failure and but there might be another accidental event. Otherwise, the innate and acquired immune system may react strongly against danger signals in case 3.

## 5. Conclusions

We reported the cytokine levels in patients infected by *R. japonica* before and after the treatment of minocycline. The current information would be beneficial to understanding the immune reaction against *R. japonica* infection especially for four data points. IL-6 and IFN-γ seem to be critical cytokines as a defense mechanism against *R. japonica* in the acute phase, and this may be linked to the decrease in eosinophils.
